# FastTagger: an efficient algorithm for genome-wide tag SNP selection using multi-marker linkage disequilibrium

**DOI:** 10.1186/1471-2105-11-66

**Published:** 2010-01-29

**Authors:** Guimei Liu, Yue Wang, Limsoon Wong

**Affiliations:** 1 Department of Computer Science, National University of Singapore, Singapore; 2 NUS Graduate School for Integrative Science and Engineering, National University of Singapore, Singapore

## Abstract

**Background:**

Human genome contains millions of common single nucleotide polymorphisms (SNPs) and these SNPs play an important role in understanding the association between genetic variations and human diseases. Many SNPs show correlated genotypes, or linkage disequilibrium (LD), thus it is not necessary to genotype all SNPs for association study. Many algorithms have been developed to find a small subset of SNPs called tag SNPs that are sufficient to infer all the other SNPs. Algorithms based on the *r*^2 ^LD statistic have gained popularity because *r*^2 ^is directly related to statistical power to detect disease associations. Most of existing *r*^2 ^based algorithms use pairwise LD. Recent studies show that multi-marker LD can help further reduce the number of tag SNPs. However, existing tag SNP selection algorithms based on multi-marker LD are both time-consuming and memory-consuming. They cannot work on chromosomes containing more than 100 k SNPs using length-3 tagging rules.

**Results:**

We propose an efficient algorithm called FastTagger to calculate multi-marker tagging rules and select tag SNPs based on multi-marker LD. FastTagger uses several techniques to reduce running time and memory consumption. Our experiment results show that FastTagger is several times faster than existing multi-marker based tag SNP selection algorithms, and it consumes much less memory at the same time. As a result, FastTagger can work on chromosomes containing more than 100 k SNPs using length-3 tagging rules.

FastTagger also produces smaller sets of tag SNPs than existing multi-marker based algorithms, and the reduction ratio ranges from 3%-9% when length-3 tagging rules are used. The generated tagging rules can also be used for genotype imputation. We studied the prediction accuracy of individual rules, and the average accuracy is above 96% when *r*^2 ^≥ 0.9.

**Conclusions:**

Generating multi-marker tagging rules is a computation intensive task, and it is the bottleneck of existing multi-marker based tag SNP selection methods. FastTagger is a practical and scalable algorithm to solve this problem.

## Background

A single-nucleotide polymorphism (SNP) is a DNA sequence variation occurring when a single nucleotide--A, T, C, or G--in the genome differs between members of a species (or between paired chromosomes in an individual). SNPs are the most common genetic variations in the human genome, and they are very important for understanding the genetic basis of common diseases. Millions of SNPs are present in human genome. The enormous number of SNPs presents a challenging problem for genome-wide association study. It has been observed that adjacent SNPs are often highly correlated. To reduce genotyping cost, many algorithms have been developed to select a smallest set of SNPs such that all the other SNPs can be inferred from them. The selected SNPs are called *tag SNPs*.

Existing tag SNP selection methods can be classified into two categories: block based methods [1-7] and genome-wide approaches [8-13]. Block based methods rely on a predefined haplotype block structure. The blocks are separated by recombination hot-spots, and there are few recombinations within a block. Thus the haplotypes within a block usually are of low diversity. They then attempt to select a subset of SNPs that can discriminate all common haplotypes within each block. The genome-wide tag SNP selection algorithms do not need to partition the whole chromosome into blocks, and they utilize linkage disequilibrium among nearby SNPs to find tag SNPs. Among the genome-wide approaches, those based on the *r*^2 ^linkage disequilibrium statistic have gained increasing popularity recently because *r*^2 ^is directly related to statistical power to detect disease associations [14].

Algorithm LD-select [9] is the first algorithm using the *r*^2 ^LD statistic to select tag SNPs, and it employs a greedy approach to find tag SNPs. Following it, several other algorithms based on the *r*^2 ^statistic have been developed. FESTA [12] breaks down large marker sets into disjoint pieces, where exhaustive searches can replace the greedy algorithm, thus leading to smaller tag SNP sets. MultiPop-TagSelect [15] and REAPER [11] apply LD-select to multiple populations. LRTag [13] uses a Lagrangian relaxation algorithm to find tag SNPs across multiple populations. All these algorithms use pairwise LD between SNPs.

Recent studies have shown that multi-marker LD can help further reduce the number of tag SNPs needed [16-18], and several algorithms have been developed to select tag SNPs based on multi-marker *r*^2 ^statistics [19-21]. These algorithms find association rules of the form {*SNP*_1_, ⋯, *SNP*_*k*_} → *SNP*_*x*_, where *k *≤ 3, *SNP*_*x *_∉ {*SNP*_1_, ⋯, *SNP*_*k*_} and the *r*^2 ^statistic between the left hand side and the right hand side of the rule is no less than a predefined threshold. Their results show that the multi-marker LD model can reduce the number of tag SNPs significantly compared with pairwise algorithms. However, existing multi-marker based algorithms are both time-consuming and memory-consuming. Most of the time is spent on calculating multi-marker *r*^2 ^statistics. Furthermore, an excess number of multi-marker association rules may be generated when *k *≥ 3, which incurs high memory consumption when using these rules to select tag SNPs. It takes hundreds of hours for the MultiTag algorithm [19,20] to finish on chromosomes containing around 30 k SNPs. The MMTagger algorithm [21] needs several hours to finish, but it consumes more than 1 GB memory. MMTagger cannot work on chromosomes with more than 100 k SNPs when *k *≥ 3. In this paper, we propose a multi-marker LD based tag SNP selection algorithm called FastTagger. FastTagger employs several techniques to reduce running time and memory consumption: (1) It merges nearby equivalent SNPs together to reduce the number of multi-marker association rules to be tested. (2) FastTagger prunes redundant rules to reduce the number of rules generated. (3) If there are too many rules, FastTagger uses a heuristics to skip some rules, that is, a rule is skipped if the right hand side of the rule has been covered enough number of times. (4) If the total size of the rules generated exceeds the memory size, FastTagger divides the chromosome into chunks, and then finds tag SNPs within each chunk. This technique can make FastTagger work on chromosomes containing more than 100 k SNPs with as less as 50 MB memory.

## Methods

In this section, we first describe how to calculate multi-marker *r*^2 ^statistics, and then present the FastTagger algorithm. The FastTagger algorithm consists of two steps. In the first step, it generates tagging rules, and in the second step, it uses a greedy approach to select tag SNPs using rules generated in the first step.

### Multi-marker tagging rules

Most SNPs have only two alleles, so we consider only bi-allelic SNPs. Given a population, the allele with higher frequency in the population is called major allele, and the allele with lower frequency is called minor allele. We use uppercase letters to denote the major alleles of SNPs, and use lowercase letters to denote the minor alleles. SNPs that are far apart from each other usually are not linked. Here we require that the distance between every pair of SNPs in a rule must not exceed a predefined distance threshold *max_dist*.

Given *k *SNPs *S *= {*SNP*_1_, *SNP*_2_, ⋯, *SNP*_*k*_}, there are 2^*k *^possible haplotypes over the *k *loci. To calculate the *r*^2 ^statistic of rule *S *→ *SNP*_*x*_, we need to divide the 2^*k *^haplotypes into two non-empty groups and map the two groups to the two alleles of *SNP*_*x*_. MultiTag [19] and MMTagger [21] uses different methods to do the mapping.

#### The one-vs-the-rest model

MultiTag uses this model. There are totally  - 2 possible ways to group the 2^*k *^haplotypes into two non-empty groups. MultiTag considers only 2^*k *^ways such that one group contains only one haplotype, and the other group contains all the other haplotypes. It calculates the *r*^2 ^statistics for all the 2^*k *^groupings, and then select the one with the highest *r*^2 ^statistic.

#### The co-occurrence model

MMTagger does the mapping based on the co-occurrences of the alleles of the SNPs on the left hand side and the alleles of the SNP on the right hand side. Let *H *be a haplotype over the SNP set *S *on the left hand side, *A *and *a *be the two alleles of *SNP*_*x *_on the right hand side, and *f*(*H*) be the frequency of *H*. We use *f*(*HA*) to denote the frequency of *H *and *SNP*_*x *_= *A *occurring together, and *f*(*Ha*) to denote the frequency of *H *and *SNP*_*x *_= *a *occurring together. If *f *(*HA*) >*f *(*Ha*), we map haplotype *H *to allele *A *of *SNP*_*x*_, otherwise we map haplotype *H *to allele *a *of *SNP*_*x*_. Let *H*_*A *_be the set of haplotypes mapped to allele *A*, and *H*_*a *_be the set of haplotypes mapped to allele *a*. We convert SNP set *S *to a bi-allelic marker with two "alleles" *H*_*A *_and *H*_*a*_. Then we can calculate the *r*^2 ^statistic between *S *and *SNP*_*x *_as follows.(1)

where *P*(*H*_*A*_), *P *(*H*_*a*_), *P *(*A*), *P *(*a*) and *P *(*H*_*A*_*A*) are the relative frequencies of *H*_*A*_, *H*_*a*_, *A*, *a *and *H*_*A*_*A *respectively.

We implemented both models in the FastTagger algorithm, and let users choose which model they want to use.

If the *r*^2 ^statistic between *S *and *SNP*_*x *_is no less than a predefined threshold *min_r*2, we say that *SNP*_*x *_can be tagged by *S*, and *R *: *S *→ *SNP*_*x *_is a *tagging rule*. With the increase of the size of *S*, the haplotypes of *S *partition the whole dataset into finer and finer groups. In an extreme case, every haplotype of *S *occurs at most once. In this case, the association between haplotypes of *S *and alleles of *SNP*_*x *_becomes unreliable. To prevent over-fitting, we put a constraint on the size of *S*. The size of *S *should not exceeds a predefined threshold *max_size*.

The *r*^2 ^statistics can be calculated from phased haplotype data directly. If the SNP data are in the form of unphased genotype data, we can use existing haplotype inference algorithms such as PHASE [22] to convert genotype data into phased haplotype data. We can also estimate *k*-marker haplotype frequencies directly from genotype data without phasing using the algorithms described in [23,24]. The second approach is used in algorithm LD-select [9].

### Generating tagging rules

To generate all the tagging rules, we need to enumerate all the SNP sets that satisfy the maximum distance constraint and maximum size constraint, and then calculate the *r*^2 ^statistics between these SNP sets and their nearby SNPs. The search space can be enormously large when the number of SNPs is large. We use several techniques to reduce the number of rules to be tested.

#### Merging equivalent SNPs

Given two SNPs *SNP*_*i *_and *SNP*_*j*_, if *r*^2^(*SNP*_*i*_, *SNP*_*j*_) = 1, which means that *SNP*_*i *_and *SNP*_*j *_can tag each other perfectly, then we say *SNPi *and *SNPj *are equivalent. Two equivalent SNPs always have the same *r*^2 ^statistics with other SNPs, thus the computation cost of the rules involving them can be shared by merging them together.

For each group of merged equivalent SNPs, a representative SNP is picked to represent this group. FastTagger generates tagging rules between representative SNPs only. The tagging rules generated in this way are called representative tagging rules. One representative tagging rule can actually represent multiple rules. Therefore, by merging equivalent SNPs, we are not only saving computation cost, but also reducing storage overhead.

Note that not every rule represented by a representative tagging rule is valid. Some of them may not satisfy the distance constraint. Equivalent SNPs that are separated by more than *max_dist *bases cannot appear in the same rule, and merging them together can produce many false rules. To reduce the number of false rules, FastTagger only merges equivalent SNPs that are within a distance of *max_dist*.

#### Pruning redundant tagging rules

If a SNP *SNP*_*x *_can be tagged by a SNP set *S*, then any rule *S' *→ *SNP*_*x *_such that *S' *is a proper superset of *S *is redundant. FastTagger generates only non-redundant tagging rules to reduce running time and memory consumption, and the definition of non-redundant rules is given as follows:

**Definition 1 (Non-redundant tagging rule) ***Given a rule S → SNP*_*x *_*such that SNP*_*x *_*can be tagged by S, if there does not exist another rule S' → SNP*_*x *_*such that S' is a proper subset of S and SNP*_*x *_*can be tagged by S', then S → SNP*_*x *_*is called a non-redundant tagging rule*.

To prune redundant rules, before calculating the *r*^2 ^statistic between *S *and *SNP*_*x*_, FastTagger checks whether there exists a subset *S' *of *S *such that *SNP*_*x *_can be tagged by *S'*. FastTagger uses a depth-first strategy to enumerate SNP sets. This search strategy is adopted from a frequent generator mining algorithm [25], and it ensures that all the tagging rules whose left hand side is a subset of *S *are generated before *S *is processed.

There can be many tagging rules generated. To speed-up the check operation, FastTagger divides the generated tagging rules into groups based on their right hand side SNP, that is, rules with the same right hand side SNP are in the same group. FastTagger then uses a hash map to index the rules in the same group, and the hashing key is the left hand side of the rules. To check whether *S *→ *SNP*_*x *_is redundant, FastTagger searches the hash map of *SNP*_*x *_for the subsets of *S*. If there is a subset of *S *in the hash map of *SNP*_*x*_, the rule is redundant; otherwise, the *r*^2 ^statistic of the rule is calculated.

#### Skipping rules

Even though merging equivalent SNPs and removing redundant tagging rules can reduce the number of tagging rules significantly, it is still possible that a large number of tagging rules are generated in the first step, which incurs high memory consumption in the second step. FastTagger uses heuristics to further reduce the number of tagging rules generated: if a SNP *SNP*_*x *_occurs at the right hand side of tagging rules enough number of times, then *SNP*_*x *_will not be considered as right hand side candidate in future rule generation. The rationale behind this heuristics is that if a SNP can be tagged by many other SNPs, then during the tag SNP selection process, the SNP has a high probability to be covered by selected tag SNPs.

### Selecting tag SNPs using a greedy approach

Finding the smallest set of tag SNPs is computationally expensive. FastTagger uses a greedy approach similar to the one proposed in [9,19] to find a near optimal set of tag SNPs.

Let *C *be the set of candidate tag SNPs, *T *be the set of tag SNPs selected, and *V *be the set of SNPs not being covered. A SNP is covered if either it is a tag SNP or it can be tagged by some SNP set *S *such that *S *⊆ *T*. Initially, *C *and *V *contain all the SNPs, and *T *is empty.

FastTagger first identifies those SNPs that do not appear at the right hand side of any tagging rules, and these SNPs must be selected as tag SNPs. FastTagger puts them into *T *and remove them from *C*. These SNPs are also removed from *V*. For the remaining SNPs in *V*, if they can be tagged by some SNP set *S *such that *S *⊆ *T*, then they are removed from *V *too.

Next, for each SNP *SNP*_*i *_∈ *C*, FastTagger finds the set of SNPs in *V *that are covered by *SNP*_*i*_. A SNP *SNP*_*j *_in *V *is covered by *SNP*_*i *_if *SNP*_*j *_is not tagged by any subsets of *T *and there exists a subset *S *of *T *such that *SNP*_*j *_is tagged by *S *∪ {*SNP*_*i*_}.

FastTagger then picks a SNP from *C *that covers the largest number of SNPs in *V *as a tag SNP. This newly picked tag SNP is put into *T *and removed from *C*. All the SNPs that are covered by it including itself are removed from *V*. This process is repeated until *V *is empty, that is, all the SNPs have been covered. In each iteration, in order to find the set of SNPs covered by every candidate tag SNP in *C*, FastTagger needs to keep the tagging rules in memory. However, the number of rules generated can be very large. It is possible that the total size of tagging rules is too large to fit into the main memory. To solve this problem, we can break the whole chromosome into several chunks such that the rules over every chunk can fit into the main memory. We then select tag SNPs within each chunk.

When selecting tag SNPs within each chunk, only those tagging rules whose SNPs all fall into this chunk are used. To also utilize the rules across chunks, we allow two adjacent chunks to have certain overlap. The length of the overlap is determined by the *max_dist *threshold. The SNPs in one chunk that are within *max_dist *bases away from the first SNP of the next chunk are included in the next chunk since they can tag or be tagged by SNPs in the next chunk. FastTagger finds tag SNPs from each chunk from left to right. The tag SNPs selected in the current chunk that also belong to the next chunk will be passed on to the next chunk as tag SNPs. Note that if the distance between two adjacent SNPs is larger than *max_dist*, then these two SNPs are used as a breakpoint even if there is enough memory. The reason being that if the distance between two adjacent SNPs is larger than *max_dist*, then the two SNPs cannot tag each other or each other's neighbors.

Using the above method, FastTagger can work on chromosomes containing more than 100 k SNPs with as less as 50 MB memory, while existing algorithm consumes more than 1 GB memory even on chromosomes containing around 30 k SNPs.

## Results and Discussion

In this section, we study the performance of FastTagger. We conducted the experiments on a PC with 2.33 Ghz Intel(R) Core(TM) Duo CPU and 3.25 GB memory running Fedora 7. All codes were complied using g++. The source codes and executable of the FastTagger algorithm can be found in Additional file 1. We obtained the datasets from HapMap release 21 http://hapmap.ncbi.nlm.nih.gov/downloads/phasing/2006-07_phaseII/phased/ and project ENCODE http://hapmap.ncbi.nlm.nih.gov/downloads/phasing/2005-03_phaseI/ENCODE/. There are 4 populations and 10 regions in the ENCODE project. Here, we report the overall results on the ten regions for each population. The results on individual regions can be found in Additional file 2. From HapMap release 21, we selected 6 chromosomes: chr1, chr2, chr3, chr19, chr21 and chr22, and used the Han Chinese plus Japanese population. Table 1 shows the number of SNPs with *MAF *≥ 5% on the datasets. In all the experiment, we set *max_dist *to 100 k, and select only those SNPs with *MAF *≥ 5%.

**Table 1:** Datasets.

datasets	#SNPs	#Rep SNPs	datasets	#SNPs	#Rep SNPs
ENCODE CEU	7,221	2,484	chr2	169,905	85,807
ENCODE HCB	6,430	2,286	chr3	135,058	71,244
ENCODE JPT	6,216	2,196	chr19	28,931	17,807
ENCODE YRI	7,963	4,408	chr21	28,914	15,644
chr1	149,716	78,893	chr22	26,595	15,553

The "#Rep SNPs" column is the number of representative SNPs with merging window size of 100 k.

### Comparison with other algorithms

The first experiment is to compare FastTagger with LRTag [13], MMTagger [21] and MultiTag [19]. LRTag uses only pair-wise LD to find tag SNPs, and it has been shown to outperform LD-select and FESTA. Hence we choose LRTag as a representative of the pairwise algorithms. MMTagger and MultiTag both use multi-marker LD to find tag SNPs. We obtained the programs from their respective authors. FastTagger used all the techniques described previously except the skipping rules technique. LRTag takes pre-computed pairwise *r*^2 ^statistics as input, so the running time of LRTag includes only tag SNP selection time. We report the results at *min_r*2 = 0.95 here, results at *min_r*2 = 0.9 and *min_r*2 = 0.8 can be found in supplementary materials. For all the four algorithms, the selected tag SNPs can cover the whole region of interest.

We first compare FastTagger with LRTag and MultiTag on using pairwise LD to find tag SNPs. Table 2 shows the running time and the number of tag SNPs selected by the three algorithms. The running time is measured in minutes. FastTagger is several times faster than LRTag even though LRTag only needs to pick tag SNPs from pre-computed pairwise *r*^2 ^statistics while FastTagger needs to compute pairwise *r*^2 ^statistics as well as selecting tag SNPs. Both algorithms are orders of magnitude faster than MultiTag. Among the three algorithms, LRTag produces the smallest number of tag SNPs, but the difference is very small. Overall, FastTagger generates 0.31% more tag SNPs than LRTag when *min_r*2 = 0.95. MultiTag generates 1.77% more tag SNPs than FastTagger when *min_r*2 = 0.95. LRTag uses a Lagrangian relaxation algorithm to select tag SNPs instead of a greedy approach used in other algorithms. That is why it generates less tag SNPs than other algorithms.

**Table 2:** Comparison of running time and number of tag SNPs selected when pairwise LD are used.

	*min_r*2	Running time (minutes)			#tag SNPs		
		FastTagger	LRTag	MultiTag	FastTagger	LRTag	MultiTag
ENCODE CEU	0.95	0.003	0.016	10.4	2144	2127	2136
ENCODE HCB	0.95	0.003	0.014	7.5	2065	2055	2061
ENCODE JPT	0.95	0.003	0.013	6.6	1996	1990	1996
ENCODE YRI	0.95	0.004	0.008	41.6	4115	4107	4109
chr1	0.95	0.076	0.242	26.2	62190	61988	63391
chr2	0.95	0.088	0.293	30.2	66026	65822	67236
chr3	0.95	0.070	0.222	25.1	55895	55713	56972
chr19	0.95	0.015	0.032	3.6	14777	14744	15014
chr21	0.95	0.015	0.040	6.0	12455	12435	12658
chr22	0.95	0.014	0.033	7.9	12690	12652	12932

The running time of LRTag includes only tag SNP selection time, while the running time of FastTagger and MultiTag includes both rule generation time and tag SNP selection time. MMTagger is excluded from this table because the MMTagger program provided by its authors cannot use pairwise LD to find tag SNPs.

Table 3 shows the running time and the number of tag SNP selected by the FastTagger, MMTagger and MultiTag when multi-marker LD are used. We implemented both models in FastTagger, and denote them as Fast-COOC (the co-occurrence model) and Fast-1vsR (the one-vs-the-rest model). MultiTag took extremely long time to finish on the 6 chromosomes when *max_size *= 3, so its results are not reported on the 6 chromosomes when *max_size *= 3. When *max_size *= 2, we divided chr1, chr2 and chr3 into 20 chunks, chr19, chr21 and chr22 into 5 chunks, and then ran MultiTag on each chunk and combined the results. MMTagger terminated abnormally on chr1, chr2 and chr3 when *max_size *= 3 because too many rules were generated. To solve this problem, we divided the three chromosomes into 10 chunks, and then ran MMTagger on each chunk and combined the results together.

**Table 3:** Comparison of running time and number of tag SNPs selected when multi-marker LD are used.

	*max_size*	*min_r*2	Running time (minutes)				#tag SNPs			
			Fast-COOC	MMTagger	Fast-1vsR	MultiTag	Fast-COOC	MMTagger	Fast-1vsR	MultiTag
ENCODE CEU	2	0.95	0.038	0.041	0.048	≥10 hours	1282	1282	1291	1371
ENCODE HCB	2	0.95	0.032	0.032	0.042	≥10 hours	1305	1328	1308	1424
ENCODE JPT	2	0.95	0.029	0.028	0.038	≥10 hours	1234	1258	1240	1349
ENCODE YRI	2	0.95	0.181	0.188	0.245	≥60 hours	2575	2618	2579	2770
chr1	2	0.95	1.13	5.84	1.40	≥7 days	43202	43483	43306	43462
chr2	2	0.95	1.32	7.21	1.63	≥7 days	44135	44556	44225	49289
chr3	2	0.95	1.14	5.11	1.41	≥7 days	37881	38206	37952	39300
chr19	2	0.95	0.176	0.343	0.218	≥30 hours	11151	11192	11160	11747
chr21	2	0.95	0.287	0.473	0.359	≥60 hours	8543	8627	8564	9103
chr22	2	0.95	0.370	0.567	0.468	≥100 hours	8970	9025	8993	9533

ENCODE CEU	3	0.95	1.28	3.69	1.85	≥50 hours	972	1017	1151	1244
ENCODE HCB	3	0.95	1.26	3.40	1.93	≥80 hours	1003	1034	1170	1170
ENCODE JPT	3	0.95	1.06	2.74	1.60	≥50 hours	958	1002	1129	1244
ENCODE YRI	3	0.95	11.6	36.7	17.4	≥14 days	1848	1927	2165	2516
chr1	3	0.95	34.9	137.3	49.6	-	35556	38185	40534	-
chr2	3	0.95	42.9	166.9	60.8	-	35502	38372	41129	-
chr3	3	0.95	39.3	154.6	55.5	-	30695	33041	35305	-
chr19	3	0.95	4.34	16.6	6.25	-	9444	10032	10546	-
chr21	3	0.95	9.91	37.7	14.4	-	6929	7404	7935	-
chr22	3	0.95	16.5	65.3	24.4	-	7327	7788	8392	-

Fast-COOC represents the FastTagger algorithm using the co-occurrence model, and Fast-1vsR represents the FastTagger algorithm using the one-vs-the-rest model. *max_size *is the maximum number of SNPs on the left hand side of a tagging rule. For the MMTagger algorithm, we divided chr1, chr2 and chr3 into 10 chunks when *max_size *= 3, and ran MMTagger on each chunk, and then combined the results. For the MultiTag algorithm, we divided chr1, chr2 and chr3 into 20 chunks, chr19, chr21 and chr22 into 5 chunks when *max_size *= 3. When *max_size *= 3, MultiTag took too long to finish on the 6 chromosomes, so we did not get its results on the 6 chromosomes.

Table 3 shows that the multi-marker model can reduce the number of tag SNPs significantly under the same *min_r*2 threshold compared with the pairwise model (Table 2). The number of tag SNPs is reduced by more than 30% when *max_size *= 2. When *max_size *= 3, the number of tag SNPs is reduced by more than 40%. However, calculating multi-marker *r*^2 ^statistics is much more expensive than computing pairwise *r*^2^. FastTagger is more than 10 times slower when *max_size *= 2, and hundreds of times slower when *max_size *= 3.

On ENCODE regions, FastTagger and MMTagger take similar time to finish when *max_size *= 2; when *max_size *= 3, FastTagger is 2-3 times faster than MMTagger. On the 6 chromosomes, FastTagger is 2-6 times faster than MMTagger. Both algorithms are orders of magnitude faster than MultiTag. The number of tag SNPs selected by FastTagger under the co-occurrence model is smaller than that selected by MMTagger and MultiTag.

Table 4 shows the maximum memory usage of FastTagger and MMTagger with *max_r*2 = 0.95 and *max_size *= 3. MMTagger consumes much more memory than FastTagger, that is why it cannot work on large chromosomes such as chr1, chr2 and chr3 when *max_size *= 3.

**Table 4:** Memory usage of FastTagger and MMTagger.

	FastTagger	MMTagger		FastTagger	MMTagger
chr1	94.41 MB	-	chr19	30.29 MB	657 MB
chr2	287.50 MB	-	chr21	74.99 MB	1210 MB
chr3	119.72 MB	-	chr22	50.20 MB	1216 MB

The co-occurrence model is used in FastTagger. *min_r*2 = 0.95, *max_size *= 3.

Table 3 also shows that the co-occurrence model generates smaller set of tag SNPs than the one-vs-the-rest model. The reason being that more rules are generated under the co-occurrence model as shown in Table 5. When *max_size *= 2, the two models generate similar number of rules, so does the number of tag SNPs. When *max_size *= 3, the co-occurrence model generates 3-4 times more rules than the one-vs-the-rest model, hence it can use much less tag SNPs to tag all the other SNPs. The co-occurrence model also consumes much more memory when *max_size *= 3 as shown in the last two columns of Table 5.

**Table 5:** The number of tagging rules generated under the two models using the FastTagger algorithm (*min_r*2 = 0.9).

	*max_size*	#rules		memory	
		Fast-COOC	Fast-1vsR	Fast-COOC	Fast-1vsR
chr19	2	121,122	120,627	6.63 MB	6.63 MB
chr21	2	169,864	168,936	11.43 MB	11.43 MB
chr22	2	156,134	155,223	8.14 MB	8.13 MB

chr19	3	1,421,519	377,773	38.69 MB	13.29 MB
chr21	3	2,713,338	657,767	101.11 MB	29.92 MB
chr22	3	2,590,826	573,738	67.28 MB	19.21 MB

### The effectiveness of the techniques used in FastTagger

This experiment studies the effectiveness of the techniques used by FastTagger in reducing running time and memory consumption. We used the co-occurrence model in this experiment because it generates more rules and is more memory demanding than the one-vs-the-rest model. The baseline FastTagger algorithm in this experiment uses two techniques as in the previous experiment: merging equivalent SNPs and pruning redundant tagging rules. The running time and memory consumption of the baseline algorithm, and the number of tag SNPs and tagging rules generated by the baseline algorithm on chr19, chr21 and chr22 when *max_size *= 3 and *min_r*2 = 0.95 is shown in Table 6.

**Table 6:** Baseline algorithm: merging equivalent SNPs and pruning redundant rules, no skipping rules.

	time	#tag SNPs	mem	#rules
chr19	4.34	9444	30.29 MB	*951,392*
chr21	9.91	6929	74.99 MB	*1,747,900*
chr22	16.5	7327	50.20 MB	*1,658,769*

The co-occurrence model is used. *max_size *= 3, *min_r*2 = 0.95.

The "#Rep SNPs" column in Table 1 shows the number of representative SNPs after merging equivalent SNPs using window size of 100 k. The number of SNPs is reduced by around a half. We have tried to use a larger window size to merge equivalent SNPs, and the results show that larger window sizes do not achieve much further reduction. The reduction in number of SNPs greatly reduces the number of rules to be tested. Table 7 shows the performance of FastTagger without merging equivalent SNPs. Without merging equivalent SNPs, FastTagger generates an excessive number of tagging rules, e.g., around 20 times more than that of merging equivalent SNPs, thus taking much longer time and consuming much more memory. There is also a slight increase in the number of tag SNPs selected.

**Table 7:** Baseline algorithm WITHOUT merging equivalent SNPs.

	time	#tag SNPs	mem	#rules
chr19	31.4	9476	209.83 MB	*17,798,798*
chr21	72.3	6959	555.42 MB	*35,278,021*
chr22	90.5	7342	340.59 MB	*30,954,495*

The co-occurrence model is used. *max_size *= 3, *min_r*2 = 0.95.

Table 8 shows the performance of FastTagger without pruning redundant rules. Pruning redundant rules can reduce the number of rules generated by 3 times, thus reducing the maximum memory usage of FastTagger by more than a half. Although identifying redundant rules can reduce the search space, it also incurs some overhead. Hence the running time of FastTagger does not decrease when it uses the pruning redundant rules technique.

**Table 8:** Baseline algorithm WITHOUT pruning redundant rules.

	time	#tag SNPs	mem	#rules
chr19	4.24	9439	75.70 MB	*3,048,090*
chr21	9.60	6942	191.86 MB	*5,643,004*
chr22	15.8	7327	130.19 MB	*5,563,473*

The co-occurrence model is used. *max_size *= 3, *min_r*2 = 0.95.

Table 9 shows performance of FastTagger when the skipping rules technique is used. Here if a SNP appears in the right hand side no less than 5 times, the SNP will not be considered as right hand side any more. By using this technique, the number of rules generated is reduced by more than a half. The running time and memory usage of FastTagger is also reduced. The number of tag SNPs selected increases slightly, but it is still smaller than that generated by the MMTagger algorithm.

**Table 9:** Baseline algorithm with skipping rules: if a SNP appears in the right hand side no less than 5 times, the SNP will not be considered as right hand side any more.

	time	#tag SNPs	mem	#rules
chr19	3.66	9550	18.61 MB	*461,139*
chr21	8.06	7086	40.74 MB	*754,084*
chr22	13.5	7447	28.62 MB	*755,309*

The co-occurrence model is used. *max_size *= 3, *min_r*2 = 0.95.

We also tested FastTagger under a memory constraint. The maximum memory can be used by FastTagger is limited to 50 MB. We used the three large chromosomes, chr1, chr2 and chr3, in this experiment. All the three chromosomes contain more than 100 k SNPs. Table 10 shows even with as less as 50 MB memory, FastTagger can still work on chromosomes with 100 k SNPs. There is only a tiny increase in its running time and the number of tag SNPs generated.

**Table 10:** Performance of Fast-COOC when memory size is restricted to 50 MB (*max_size *= 3, *min_r*2 = 0.95)

	No memory constraint			mem = 50 MB		
	time	#tag SNPs	mem	time	#tag SNPs	#chunks
chr1	34.9	35556	94.41 MB	35.14	35561	16
chr2	42.9	35502	287.50 MB	43.14	35518	21
chr3	39.3	30695	119.72 MB	39.3	30706	15

### Portability and prediction accuracy

Multi-marker models group combinations of the alleles on the left hand side into two groups, and then map these two groups to the two alleles on the right hand side. Compared with pairwise model, multi-marker models are more prone to over-fitting. Here we use three populations in HapMap--the Han Chinese population (HCB), the Japanese population (JPT) and the Caucasian population(CEU)--to study the portability and prediction accuracy of tagging rules of different lengths. We use chr19 in this experiment. We first generate tagging rules from one population, and then calculate the *r*2 statistics and prediction accuracy of these rules in the other populations. The prediction accuracy of a rule is defined as the proportion of alleles of the SNP on the right hand side that are correctly predicted by the alleles of the SNPs on the left hand side. The results reported below are results when rules are generated from individuals in the Han Chinese population and are evaluated using individuals in the other two populations. In all three populations, we consider only those SNPs with MAF ≥ 5%.

**Figure 1:**
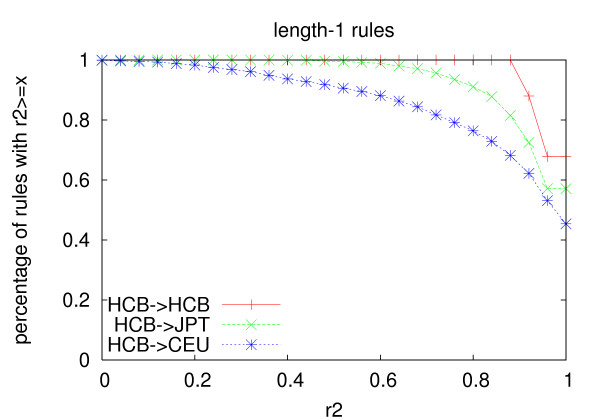
**Portability of length-1 rules**. The rules are generated from the Han Chinese population with *min_r*2 = 0.9, and they are then validated on the other two datasets as well.

**Figure 2:**
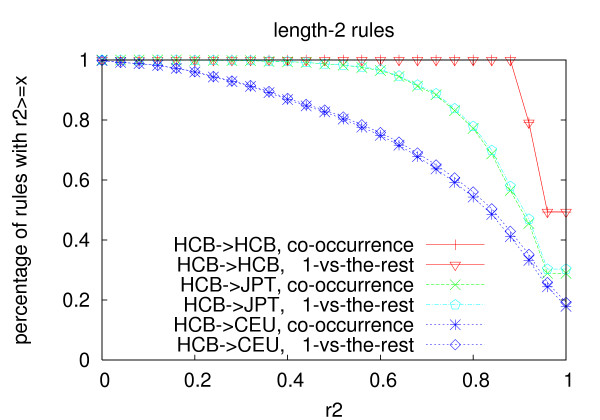
**Portability of length-2 rules**. The rules are generated from the Han Chinese population with *min_r*2 = 0.9.

**Figure 3:**
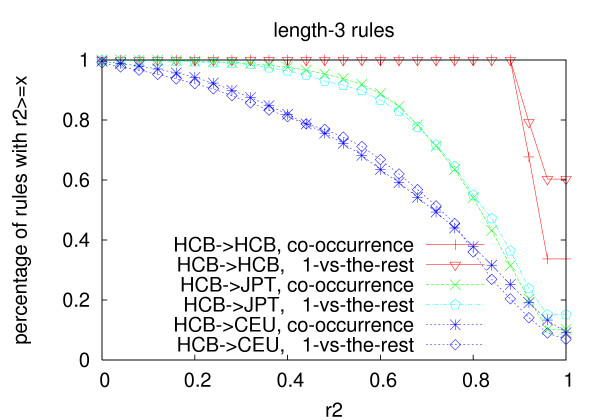
**Portability of length-3 rules**. The rules are generated from the Han Chinese population with *min_r*2 = 0.9.

Figures 1, 2 and 3 show the distribution of the *r*^2 ^values of the rules generated from the Han Chinese population using the two multi-marker models in the three populations. Table 11 shows average *r*^2 ^of the rules in the three populations. For all the three lengths, the average *r*^2 ^of the rules in the Japanese population and the Caucasian population is lower than that in the Chinese population. The decrease of length-2 and length-3 rules is more significant than that of length-1 rules, which indicates that longer rules are more prone to over-fitting than shorter rules for both models. The *r*^2 ^values of the rules become much lower in the Caucasian population than that in the Japanese population, which is consistent with the genetic differences between the three populations.

**Table 11:** Average *r*^2 ^and predication accuracy of rules of different length on three populations.

		#rules			average *r*^2^			average accuracy		
len	model	HCB	JPT	CEU	HCB	JPT	CEU	HCB	JPT	CEU
1	pairwise	85961	84123	69083	0.978	0.942	0.865	0.995	0.989	0.966

2	co-occurrence	1563176	1472654	1014934	0.965	0.878	0.745	0.993	0.977	0.938

2	one-vs-the-rest	1560181	1469765	1012699	0.965	0.881	0.753	0.993	0.977	0.940

3	co-occurrence	26182522	24495802	16064120	0.952	0.790	0.665	0.990	0.960	0.913
3	one-vs-the-rest	7074493	6269985	3955224	0.970	0.791	0.659	0.994	0.970	0.919

The rules are generated from Han Chinese population with *min_r*2 = 0.9. Some rules may become invalid in the other two populations because the MAF of some SNPs in the other two populations may be smaller than 5%. When only pairwise LD is used, all algorithms generate the same set of rules. When multi-markers are considered, FastTagger-COOC and MMTagger generate the same set of rules using the co-occurrence model; FastTagger-avsR and MultiTag generate the same set of rules using the one-vs-the-rest model.

**Figure 4:**
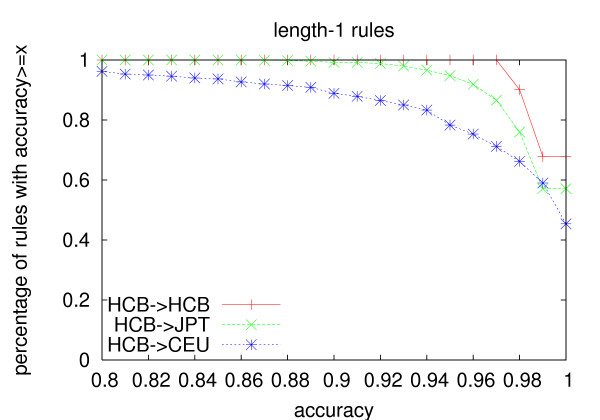
**Prediction accuracy of length-1 rules**. The rules are generated from the Han Chinese population with *min_r*2 = 0.9.

**Figure 5:**
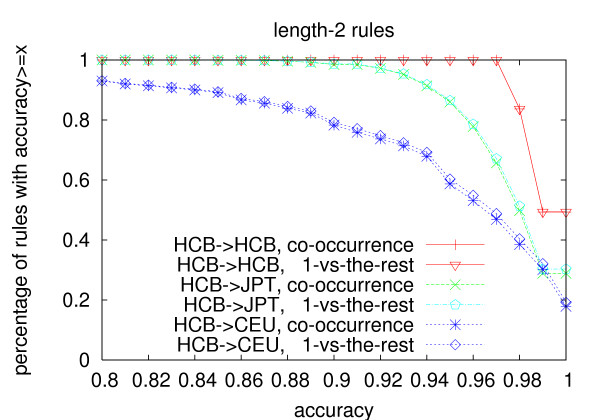
**Prediction accuracy of length-2 rules**. The rules are generated from the Han Chinese population with *min_r*2 = 0.9.

**Figure 6:**
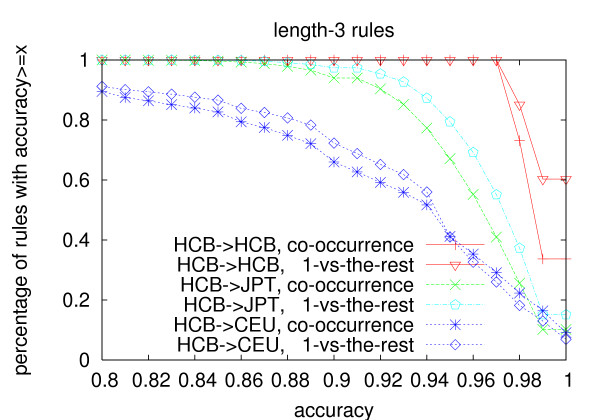
**Prediction accuracy of length-3 rules**. The rules are generated from the Han Chinese population with *min_r*2 = 0.9.

The same trend is observed on prediction accuracy (Figure 4, 5 and 6). Even though the rules are generated from the Chinese population, their accuracy in the Japanese population is always above 80%. Even for length-3 rules, 94% rules generated using the co-occurrence model have an accuracy no less than 90%, and 97.4% rules generated using the one-vs-the-rest model have an accuracy no less than 90% in the Japanese population. The average accuracy of length-3 rules is above 96% for both models in the Japanese population(Table 11). The average accuracy of the rules in the Caucasian population is lower than that in the Japanese population, but it is still above 91% even for length-3 rules. We believe that if we use individuals from the same population to do the testing, the average *r*^2 ^and accuracy should be even higher. As for the two models, the number of length-2 rules generated by the two models is similar, while the co-occurrence model generates about 3.5 times more length-3 rules than the one-vs-the-rest model. The average *r*^2 ^and accuracy of the length-3 rules generated using the one-vs-the-rest model is higher than that generated using the co-occurrence model on both populations. However, since much less rules are generated under the one-vs-the-rest model, the one-vs-the-rest model needs more tag SNPs to cover all the other SNPs than the co-occurrence model as shown in Table 3.

## Conclusions

In this paper, we have presented an efficient algorithm called FastTagger for genome-wide tag SNP selection using multi-marker LD. FastTagger uses several techniques to reduce running time and memory consumption. Our experiment results show that FastTagger is several times faster than existing tag SNP selection algorithms using multi-marker models, and it consumes much less memory at the same time, which makes FastTagger can work on chromosomes containing more than 100 k SNPs where existing algorithms using multi-marker models usually fail. FastTagger also select less tag SNPs than existing algorithms using multi-marker LD. Our experiment results also show that merging equivalent SNPs together is the most effective technique in reducing running time and memory consumption.

We implemented two multi-marker models in the FastTagger algorithm. The one-vs-the-rest model generates rules with higher average *r*^2 ^and higher average accuracy than the co-occurrence model under the same parameter settings. However, it generates much less length-3 rules than the co-occurrence model, thus requiring more tag SNPs to cover all the other SNPs.

We compared the portability and prediction accuracy of rules of different length. The results show that shorter rules have better portability and higher prediction accuracy than longer rules. Nevertheless, length-3 rules generated from the Chinese population can still achieve an average accuracy of 96% on the Japanese population for both models.

In our experiments, we calculate prediction accuracy for individual rules. When we use these rules to make predictions on unobserved SNPs, it is possible that one SNP can be predicted by multiple rules, and the prediction of these rules may conflict with one another. In our future work, we will study how to resolve the conflicts and make consensus predictions for unobserved SNPs.

## Availability and requirements

• **Project name**: Pattern spaces & data mining algorithms for pharmacogenomics

• **Project home page**: http://www.comp.nus.edu.sg/~wongls/projects/snp-analysis/index.html

• **Grant**: A*STAR SERC PSF 072-101-0016

• **Operating system(s)**: Linux or Windows

• **Programming language**: C++

• **Other requirements**: none

• **License**: FreeBSD for academic use

• **Any restrictions to use by non-academics**: Licence needed

## Authors' contributions

Guimei Liu designed and implemented the FastTagger algorithm, and wrote this manuscript. Yue Wang participated in discussion of the proposed method and conducted the experiments. Limsoon Wong gave advice on the design of the algorithm and the manuscript. All authors read and approved the final manuscript.

## Supplementary Material

Additional file 1 File "FastTagger.zip" contains the source codes and executables of the FastTagger program, both for Linux and Windows. Please read file "FastTagger.readme" on how to use the program. Click here for file

Additional file 2 File "FastTagger-sup.xls" contains additional experiment results, and it is a Microsoft Excel file. Click here for file
